# Mental Health Problems in Family Medicine/General Practice

**DOI:** 10.1155/2012/794845

**Published:** 2012-10-03

**Authors:** Jan De Lepeleire, Marian Oud, Marta Buszewicz

**Affiliations:** ^1^Department of Public Health and Primary Care, KU Leuven, Kapucijnenvoer 33, Blok J, 3000 Leuven, Belgium; ^2^Universitair Medisch Centrum Groningen, Groningen, The Netherlands; ^3^Research Department of Primary Care and Population Health, University College London, London NW3 2PF, UK

Mental health disorders represent a large proportion of the world's disease burden, mostly due to depression and anxiety disorders, alcohol and drug abuse, and psychoses [1, 2].

In terms of disability-adjusted life years, depression ranks number one among noncommunicable diseases and is projected to become the most important overall contributor to the global disease burden by 2030 [3]. 

People with mental problems usually seek help from their general practitioner (GP), sometimes in combination with a psychiatrist [4]. In this current period of economic recession, delivering health care in an efficient and cost-effective way is particularly important. Primary health care teams are being asked to take on increased responsibilities in the implementation of strategies to help in the prevention and treatment of mild and moderate mental health problems and effective coordination with secondary care for more severe conditions.

Mental health disorders are very often comorbid with physical health disorders [5]. They can play an important role in the development of somatic diseases and vice versa. The burden of mental disorders is likely to be underestimated as a consequence of the inadequate recognition of the connection between mental and physical health. Indeed, as mental and physical health are correlated, there can be no health without mental health [2]. 

In this special mental health edition of the International Journal of Family Medicine we are pleased to present a selection of papers from 5 different European countries, looking in more depth at some of these important issues.

E. M. McMahon et al. investigated the functional impairment, service use, and costs of patients with chronic and recurrent depression recruited from UK primary care. These patients visited their GP more often than other patients and were also high users of hospital-based services (including both physical and mental health care), especially those patients with chronic major depression. This was a patient group with very significant morbidity and high costs, highlighting the need for effective interventions. A. Wallerblad et al. found that forty-seven percent of those affected by mild depression and/or anxiety had been seeking care for psychological symptoms within the last year. Those who had consulted their GP about their psychological problem and those who had also seen a psychiatrist, psychologist, or alternative health provider were more likely to have both depression and anxiety, and disability due to their psychological symptoms and so likely to be more severely affected. Among those not seeking care for their psychological symptoms, two-thirds had sought care for somatic symptoms, which might be linked with them, focusing on the physical symptoms which often accompany depression and anxiety and highlight the importance of GPs actively, also considering mental health problems in patients who present with physical symptoms [6]. 

How these and other determinants influence pathways of care and referral from primary care physicians (PCPs) to a Community Mental Health Centre (CMHC) in the context of a collaborative care programme for common mental disorders is discussed by P. Rucci et al., working in Bologna, Italy. In their study, of 8,570 patients diagnosed as having common mental health problems, 57.4% were referred by PCPs to CMHCs. Those less likely to be referred were living in urban areas, suffered from depression rather than anxiety, and were younger. However, once they had been referred and assessed, patients living in urban areas were more likely to receive “shared care”, with the consultant providing brief and focused therapeutic interventions to support the PCP management of psychiatric disorders. Prospective studies are needed to assess the optimum length, quantity, and quality of collaborative treatment to improve outcomes for common mental disorders delivered at any step of the care pathway.

A potential adverse consequence of undetected mental health problems is prolonged absence from work, with significant costs both for the individual and society. The two-phase study by H. J. Soegaard et al. from Denmark involved screening individuals classified as being on long-term sickness absence for undetected common mental health problems not given as a reason for their sick leave. Using the Present Sate Examination (PSE) with a subsample and extrapolating the results they found the frequencies of undetected mental disorders among sick-listed individuals to be 21% for any psychiatric diagnosis, with 14% diagnosed as having depression, 4% anxiety, and 6% somatoform disorder. Clearly it is very important to detect mental health problems in this population, in order to initiate the appropriate treatment and facilitate people's return to work.

Finally, work from R. Ettner et al. from the Netherlands looks at the other side of the equation mentioned at the beginning of this editorial—how chronic stress or emotional difficulties may impact adversely on peoples' physical health. The subjects were middle-aged genetic males, of which the group being investigated were gender dysphoric and seeking treatment for this, while the controls were normal males with no gender difficulties. Gender dysphoria is an uncomfortable and stressful situation which these subjects are likely to have experienced for several years before presenting for treatment. The groups did not differ in average BMI, alcohol intake, or smoking behavior and all subjects were of middle to upper class socioeconomic status, but the mean blood pressure readings in the gender dysphoric group were significantly higher than in the controls, adding to the evidence for a significant and positive association between measures of chronic stress and hypertension. 

Coping with mental health problems is a major challenge for patients and their carers, physicians, the health care system, and the community. These interesting papers have highlighted current issues of under-detection and variable pathways for the care of people with common mental health problems in a range of European countries, but there remains a considerable challenge to improve access to health care, adequate and timely diagnoses, and cost-effective and appropriate treatment of all people with mental health problems.

*Jan De Lepeleire* *Jan De Lepeleire* *Marian Oud* *Marian Oud* *Marta Buszewicz* *Marta Buszewicz*

## References

[1] World Health Organisation (2008). *The Global Burden of Disease: 2004 Update*.

[2] Prince M, Patel V, Saxena S (2007). No health without mental health. *The Lancet*.

[3] Mathers CD, Loncar D (2006). Projections of global mortality and burden of disease from 2002 to 2030. *PLoS Medicine*.

[4] Codony M, Alonso J, Almansa J (2009). Perceived need for mental health care and service use among adults in Western Europe: results of the ESEMeD Project. *Psychiatric Services*.

[5] Von Korff M, Scott K, Gurejej O (2009). *Global Perspectives on Mental-Physical Comorbidity in the WHO World Mental Health surveys*.

[6] Tylee A, Walters P (2007). Underrecognition of anxiety and mood disorders in primary care: why does the problem exist and what can be done?. *Journal of Clinical Psychiatry*.

